# Cyclin dependent kinase 4 and 6 inhibitors as novel therapeutic agents for targeted treatment of malignant mesothelioma

**DOI:** 10.18632/genesandcancer.138

**Published:** 2017-03

**Authors:** Navid Sobhani, Silvia P. Corona, Fabrizio Zanconati, Daniele Generali

**Affiliations:** ^1^ Department of Medical, Surgery & Health Sciences, University of Trieste, Trieste, Italy; ^2^ Department of Radiation Oncology, Peter MacCallum Cancer Center, Moorabbin Campus, Australia

**Keywords:** malignant Mesothelioma, CDKN2A, inhibitor of Cyclin-Dependent Kinases 4 and 6

## Abstract

Malignant Mesothelioma (MM) is a rare and aggressive form of tumour that affects the lining of the internal organs for which current treatments have not been proven to be very effective. P16^INK4A^ tumour suppressor encoding CDKN2A gene is often downregulated in MM. This protein is a cyclin dependent kinase 4 and 6 inhibitor, that normally phosphorylates RB1, which has to be un-phosphorylated in order to block cell-cycle at G1 in normal cells. Adding CDK inhibitor molecules to MM in pre-clinical studies has been proven to restore the normal function of p16^INK4A^, blocking thereby MM cell cycle at G1. Future randomised phase III studies with CDK4/6 inhibitors in MM carrying relevant CDK4/6, cyclin D1/3 or p16 aberrations will be warranted.

## INTRODUCTION

Malignant Mesothelioma (MM) is a rare and aggressive disease, for which current treatments have not proven very effective. This tumour is particularly common in certain geographic regions with historical exposure to asbestos and according to the latest data from the Italian Registry of Tumours Association, the area of Trieste falls into the “high incidence” regions [1]. Based on data from the literature, *CDKN2A* and its protein, tumour suppressor p16^INK4a^, could be an interesting target to pursue for medical treatment, as often down-regulated in MM [2]. P16^INK4a^ (p16) protein is involved in the regulation of the cell cycle machinery, functioning as an inhibitor of Cyclin-Dependent Kinases 4 and 6 (CDK4/6). In accordance with previous reports, two recent studies found that 52.2% and 45% of pleural MMs showed deletions in the 9p21 area containing *CDKN2A* and *CDKN2B* genes [3,4]. Further confirmation of the important role that cell cycle dysregulation plays in MM was provided by Jennings *et al*.,: their research showed the presence of direct correlation between p16 protein expression levels and response to chemotherapy and therefore survival in patients with MM [5].

With the advent of selective anti-CDK 4/6 inhibitors such as palbociclib or ribociclib, already approved by FDA in the metastatic breast cancer setting, currently at different stages of clinical development, dysregulation of the cell cycle has become targetable [6]. Cyclin-dependent kinases 4 and 6, in a complex with Cyclin D, promote cell-cycle progression from G1 to S by inactivating the tumour suppressor Retinoblastoma-associated protein (RB1) and the RB1-like proteins 1 and 2. Cyclin-dependent kinase inhibitors, such as p16, also participate in the regulation/inhibition of CDK 4/6 activity. Loss of p16 and loss of RB1 function are generally mutually exclusive in cancer cells. When *CDKN2A* is mutated or hypermetylated, inhibition of CDK4/6 by p16 is lost and pro-mitotic signals prevail. In the same fashion, when RB1 is mutated, cyclin E1 and CDK2 are constitutively activated, with the initiation of transcription of pro-mitotic genes. In the latter case, cancer cells do not rely on CDK4/6 for the transition from G1 to S and therefore are not sensitive to CDK4/6 inhibitors. It is clear how both p16 and cyclin-dependent kinase 4/6 require a functional RB1 to exert their cell-cycle regulatory function. Therefore, patients selection on the basis of these molecular features becomes paramount to the success of future clinical trials investigating functional roles of CDK4/6 inhibitors in the treatment of MM. Of note, the importance of the p16 status for the diagnosis of MM was recently confirmed by Nabeshima and colleagues [2], while further studies to validate its role as a predictor of prognosis and response/resistance to treatment in patients with MM and other solid tumours are underway [7]. On the basis of the role of tumour suppressor gene/protein p16 in the cell-cycle signalling pathway, and in view of the prevalence of mutations found in MM, Frizelle S.P. *et al.* used a p16 peptide (84-103 aa), which exerts minimal CDK-inhibitory activity, to transduce mesothelioma cells to inhibit CDK4\6 and evaluated its therapeutic potential in mesothelioma. The p16 peptide (TAT-p16) expression over three MM cell lines (H2452, H2052 and H2461) induced RB1 phosphorylation and consequent cell cycle arrest at G1, followed by cell death. To corroborate these encouraging *in vitro* results, xenografts with p16-deficient mesothelioma cell line H2373 provided *in vivo* proof that induction of TAT-p16 decreases CDK4\6 activity resulting in hypophosphorylation of RB1 [8]. Treatment of mesothelioma with p16 adenovirus was previously demonstrated a useful therapeutic approach in other two *in vivo* studies by the same authors [9,10].

Another proof-of-concept supporting the anti-tumour activity of p16-mimicking molecules comes from a patent filed for novel CDK4/6 inhibitors for the treatment of malignant mesothelioma, where the authors showed that the new inhibitors are able to stop proliferation of three MM cell lines (namely MSTO-211, NCI-H2052, NCI-H28) *in vitro* with cytostatic activity in the micromolar range [11].

After such promising results, a phase II clinical trial has been designed to evaluate the CDK 4/6-inhibitor ribociclib in solid tumours, including MM carrying relevant CDK4/6, cyclin D1/3 or p16 aberrations (NCT02187783).

Our report would support the need for randomized phase III clinical studies specifically designed to test the anti-tumour activity of CDK4/6 inhibitors as single agents or in combination in MM on the basis of the molecular characteristics of this tumour.
